# Mapping the Transglycosylation Relevant Sites of Cold-Adapted β-d-Galactosidase from *Arthrobacter* sp. 32cB

**DOI:** 10.3390/ijms21155354

**Published:** 2020-07-28

**Authors:** Maria Rutkiewicz, Marta Wanarska, Anna Bujacz

**Affiliations:** 1 Institute of Molecular and Industrial Biotechnology, Faculty of Biotechnology and Food Sciences, Lodz University of Technology, Stefanowskiego 4/10, 90-924 Lodz, Poland; anna.bujacz@p.lodz.pl; 2 Macromolecular Structure and Interaction, Max Delbrück Center for Molecular Medicine, Robert-Rössle-Straße 10, 13125 Berlin, Germany; 3 Department of Molecular Biotechnology and Microbiology, Faculty of Chemistry, Gdansk University of Technology, Narutowicza 11/12, 80-233 Gdansk, Poland; marta.wanarska@pg.edu.pl

**Keywords:** β-d-Galactosidase, cold-adapted, transglycosylation, lactulose, sucrose, complex structures, crystal structures, mutants

## Abstract

β-Galactosidase from *Arthrobacter* sp. 32cB (*Arth*βDG) is a cold-adapted enzyme able to catalyze hydrolysis of β-d-galactosides and transglycosylation reaction, where galactosyl moiety is being transferred onto an acceptor larger than a water molecule. Mutants of *Arth*βDG: D207A and E517Q were designed to determine the significance of specific residues and to enable formation of complexes with lactulose and sucrose and to shed light onto the structural basis of the transglycosylation reaction. The catalytic assays proved loss of function mutation E517 into glutamine and a significant drop of activity for mutation of D207 into alanine. Solving crystal structures of two new mutants, and new complex structures of previously presented mutant E441Q enables description of introduced changes within active site of enzyme and determining the importance of mutated residues for active site size and character. Furthermore, usage of mutants with diminished and abolished enzymatic activity enabled solving six complex structures with galactose, lactulose or sucrose bounds. As a result, not only the galactose binding sites were mapped on the enzyme’s surface but also the mode of lactulose, product of transglycosylation reaction, and binding within the enzyme’s active site were determined and the glucopyranose binding site in the distal of active site was discovered. The latter two especially show structural details of transglycosylation, providing valuable information that may be used for engineering of *Arth*βDG or other analogous galactosidases belonging to GH2 family.

## 1. Introduction

β-d-Galactosidases (βDGs), enzymes catalyzing hydrolysis of β-d-galactosides, belong to five different glycosyl hydrolase (GH) families: GH1, GH2, GH35, GH42, and GH59, as the classification was made based on protein fold not its primary activity. Their common feature is the presence of a TIM barrel fold catalytic domain; however, they differ in the number of surrounding domains possessing β architecture [1].

β-d-Galactosidases belonging to the GH2 family are large proteins, usually ~100 kDa, of which the active forms are: tetramers [2,3,4], hexamers [5], and as recently discovered dimers [6,7]. Their catalytic site can be divided into a galactose binding subsite and a platform for weak binding of different moieties [8]. Their primary mode of action is to catalyze the hydrolysis of lactose to d-galactose and d-glucose. Some of GH2 β-d-galactosidases are able to catalyze transglycosylation reactions when the galactosyl moiety is transferred onto an acceptor larger than a water molecule. The best studied example is *lacZ* β-d-galactosidase from *Escherichia coli*, which produces allolactose, disaccharide composed of d-galactose, and d-glucose moieties linked through a β-(1→6)-glycosidic bond, if excess of galactose occurs [9,10].

Prebiotics are non-digestible food ingredients that support development and functioning of human organisms through selective assistance of growth or activity of one or a number of bacteria species in the lower intestine [11,12,13]. Ones that are used to enrich a daily diet are chosen to stimulate the growth of bacteria from *Bifidobacteria* or *Lactobacilli* families [14,15,16,17]. The importance of prebiotics, especially galactooligosaccharides, as an additive to infant formula has been widely tested. They support the colonization of intestines by beneficial bacteria. In consequence, they strengthen immunity by prevention of bacterial adhesion at an early stage of infection [18,19,20,21,22,23,24,25]. Morerover, some oligosaccharides are a rich source of sialic acid, indispensable for proper development of the brain [26,27]. Prebiotics are also important in adults’ nutrition, as they may support absorption of minerals [28,29,30], support recovery after influenza, reduce stress-related digestive problems [31], support lipid metabolism, and also counteract the development of tumors. They augment prevention in liver encephalopathy, glycemia/insulinemia, and also have a positive effect on immunomodulation [12,32].

Even though prebiotics such as galactooligosaccharides (GOS) and heterooligosaccharides (HOS) have been successfully synthesized, their production in the course of enzymatic catalysis proved to be more beneficial due to the higher specificity of product and milder reaction conditions. For this purpose, glycosyltransferases (EC 2.4) or glycosidic hydrolases (EC 3.2.1) are used—enzymes that have the ability to catalyze the transfer of a galactosyl moiety to a sugar acceptor.

Nowadays, both for lactose removal and GOS/HOS synthesis, the β-d-galactosidase from *Kluyveromyces lactis* is predominantly used [33,34,35,36]. Introduction of new enzyme into food production must be preceded by a number of studies that will not only ensure its high efficiency but will also ensure the consumers’ safety. Enzyme immobilization may be the way to ensure that it will not induce allergic reactions in humans.

Nonetheless, an implementation of cold-adapted enzymes would reduce the overall process temperature bringing numerous benefits [37,38]. Lowering the temperature of the process eliminates need for heating. Therefore, not only are costs being cut, but the process becomes more environmentally friendly. Furthermore, final product quality may be improved, as conducting enzymatic reactions at low temperature in food processing prevents the loss of valuable substances and formation of undesired products of heat conversions. What is more, cold-adapted enzymes are perfect candidates to perform catalysis in organic solvent environment which is especially interesting for the pharma industry.

This broad range of benefits that can be achieved by implementation of cold-adapted enzymes provides rationale for the efforts to engineer enhanced activity cold-adapted enzymes tailored for an exact industrial applications [39,40]. The natural way of research is to provide as a first step structural information that can be further used for knowledge base enzyme engineering. However, due to the difficulty of cold-adapted enzymes’ crystallization the amount of crystal structures available in Protein Data Bank is surprisingly low. Only structures of 11 cold-adapted glycosyl hydrolases are deposited there: α-amylase from *Alteromonas haloplanctis* [41], endo-1,4-beta-glucanase from *Eisenia fetida* [42], psychrophilic endo-beta-1,4-xylanase [43], β-1,4-d-mannanase from *Cryptopygus antarcticus* [44], chitinase from *Moritella marina* [45], endo-beta-1,4-xylanase from *Aegilops speltoides* subsp. speltoides [43], β-glucosidase from *Exiguobacterium antarcticum* B7 [46], xylanase from *Pseudoalteromonas haloplanktis* [47], β-galactosidase from *Rahnella* sp. R3 [48], β-d-galactosidase from *Paracoccus* sp. 32d [6], and further discussed here β-d-galactosidase from *Arthrobacter* sp. 32 cB [7]. Hence, detailed information on the structure of β-d-galactoside processing cold-adapted enzyme that could be used for prebiotics production at the industrial scale is still in demand. The modification of transglycosylase activity specificity and efficiency may be achieved by controlling reaction equilibrium or by enzyme engineering. Studies concentrated on introducing mutations into subsites of GHs showed that the modulation of hydrolysis and transglycosylation activities can be achieved by means of knowledge-based enzyme engineering. However, the role of individual amino acids in the active site must be discovered as basis for successful design of an enzyme with specific, desired activities [49].

Psychrophilic β-d-galactosidase from *Arthrobacter* sp. 32cB is an interesting candidate for industrial use, as it can hydrolyze lactose at rate comparable to mesophilic βDG from *Kluyveromyces lactis.* Furthermore, it exhibits additional transglycosylation activity, it catalyzes synthesis of GOS and HOS, for example: lactulose and alkyl glycosides. [50] Architecture of its five domains [7], structural details of hydrolysis of lactose catalyzed by this enzyme [51], and its structural features responsible for the enzyme’s cold adaptation [52] were investigated and discussed previously. Upon the solution of native structure, E441 and E517 were assigned as catalytic residues based on superposition of *Arth*βDG structure with *lacZ E. coli* β-d-galactosidase [7]. The role of residue E441 was previously proven: a loss-of-function mutant E441Q was designed, a biochemical activity assay was performed, and the protein was crystallized. The introduction of point mutation E441Q did not disrupt the structure of protein, yet no catalytic activity was exhibited anymore. The complex structures *Arth*βDG_LACs and *Arth*βDG_LACd, with natural substrate-lactose bound in shallow and deep mode revealed residues directly involved in the binding of galactosyl group in two modes [51]. Especially interesting was the D207 residue, as it contributes in the deep binding of substrate by stabilizing O4 hydroxyl group but also creates a bottom of the active site limiting its size. The question of the effect of point mutation of the other catalytic residue, E517, has not been answered before. Similarly, structural elements implicated in the enzyme’s secondary activity, transglycosylation, remain elusive.

This is why, two new mutants, *Arth*βDG_D207A and *Arth*βDG_E517Q, were designed to elucidate the impact of these two residues on protein’s structure and activity. Furthermore, here we attempted to shed a light on the structural side of transglycosylation catalyzed by cold-adapted GH2 *Arth*βDG through usage of the loss and depletion of the function mutants for crystallization and formation of complexes with selected reaction substrates or products, especially lactulose.

## 2. Results and Discussion

### 2.1. Activity of ArthβDG Mutants

The hydrolytic activity of *Arth*βDG_D207A and *Arth*βDG_E517Q was measured using ONPG as a substrate. The *Arth*βDG_E517Q exhibited no catalytic activity, whereas activity of mutant *Arth*βDG_D207A was severely depleted (Table 1).

The results of the activity assays show that the hydrolytic activity of the enzyme was affected not only when catalytic residues were mutated but also when the mutation of the amino acids that play a key role in building the active site was introduced. As expected of the mutation of the catalytic residue, E517 into glutamine caused loss of function of the enzyme. The mutation of the residue involved in the stabilization of galactosyl moiety within active site, D207, diminished the enzyme’s activity.

Such a drastic drop of activity related to the mutation of one of the residues contributing to binding and positioning of the substrate in the active site of enzyme suggests that the net of interactions within the *Arth*βDG’s active center was in a very delicate balance. This cold-adapted enzyme was already characterized by a high turnover rate, and losing contacts within its active site cripples its activity instead of further enhancement.

### 2.2. Thermofluor Shift Assay

Results of the TSA (thermofluor shift assay) of *Arth*βDG_D207A did not exhibit significant differences compared with ones obtained for wild-type protein *Arth*βDG [52]. The highest melting temperature (44 °C) was obtained in conditions containing 50 mM sodium phosphate pH 6.0. Similar stability was obtained for protein samples in buffers, such as PIPES, HEPES, MES BIS-TRIS propane, ADA, and MOPS, within a pH range 6.0–6.7, independently, on addition of 250 mM NaCl. Using the same set of buffers in the pH ranges 5.0–6.0 and 7.0–8.0 the melting temperature was on average 3 °C lower. Also, the stabilization effect of previously established for *Arth*βDG crystallization solution (37% Tascimate^TM^ pH 8.0) augments stability of *Arth*βDG_D207A by 7 °C compared to 50 mM sodium phosphate pH 6.0 (Figure S1).

Mutation of catalytic residue E517 into glutamine resulted in the elevation of the melting temperature of the mutant in respect to *Arth*βDG by 3.5–5 °C, depending on the investigated buffer solution. In the case of 50 mM sodium phosphate pH 6.0, it attained 49 °C, which means that it was higher by 4.5 °C compared to wild-type protein. The trend of increasing the melting temperature of the mutant *Arth*βDG_D207A by approximately 4 °C was maintained for all tested buffers in the pH range 6.0–7.0. In the pH ranges 5.0–6.0 and 7.0–8.0, the melting temperature was elevated on average by 3 °C compared to *Arth*βDG. Nonetheless, the impact of crystallization solution on protein stability compare to 50 mM sodium phosphate pH 6.0 was higher than in the case of *Arth*βDG_N440D, by 5 °C (Figure S2).

The results of TSA showed that the introduced mutation did not lead to a dramatic drop of mutant’s melting temperature that would be expected if mutations would have a destabilizing effect on the protein’s structure. It also showed that they exhibited similar features to wild-type protein; thus, crystallization was attempted in conditions previously established for wild-type protein without initial screening. Such an approach allowed us to obtain mutants’ crystal almost readily without need for much of a protein sample.

### 2.3. ArthβDG Mutants

**Figure 1:**
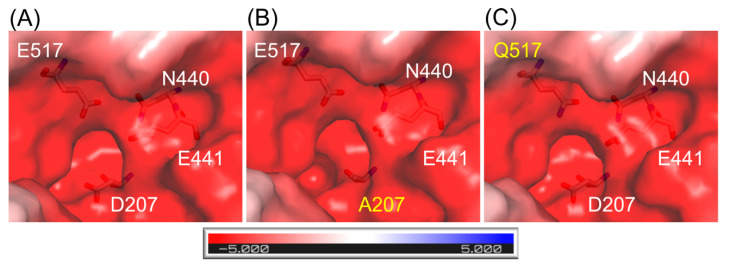
Representation of surface and charge distribution within the active site of *Arth*βDG (**A**) and its mutants: *Arth*βDG_D207A (**B**) and *Arth*βDG_E517Q (**C**). The residues under scrutiny are shown as stick and mutations are indicated with yellow. The charge distribution was calculated using the APBS [53] plugin to PyMOL.

The structures of *Arth*βDG mutants: *Arth*βDG_D207A and *Arth*βDG_E517Q, were solved proving that the point mutations within the active site of *Arth*βDG have no destabilizing effect on protein’s structure. In the case of *Arth*βDG_E517Q, only a slight change in character of the active site was observed (Figure 1C)—similar to previously studied loss of function mutant *Arth*βDG_E441Q [51].

In the case of mutant *Arth*βDG_D207A, where a larger side chain was substituted with alanine residue, the change in active site size and shape was introduced (Figure 1B).

### 2.4. Influence of Mutations within Active Site on Binding of Galactosyl Residue

The functional dimer of *Arth*βDG has two independent active sites. Each of them is located on the top of TIM-barrel Domain 3 and is constituted of residues from both subunits of the dimer. It can be described as a deep acidic funnel with an antechamber.

**Figure 2:**
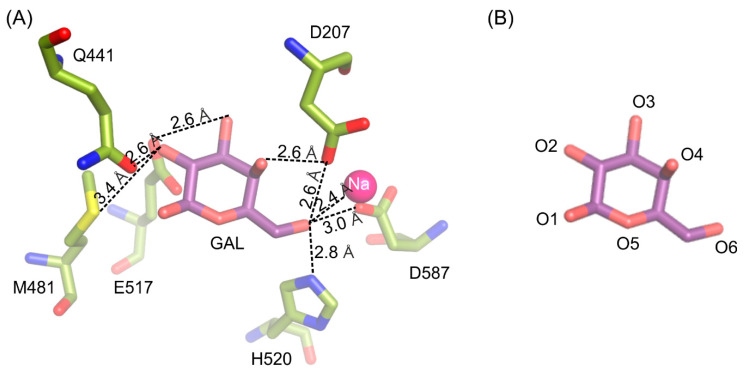
Interactions stabilizing galactose molecule in the active site of *Arth*βDG_E441Q/gal complex structure (**A**). Naming convention of galactose’s oxygen atoms presented for easier interpretation (**B**).

The galactosyl moiety is stabilized in the deep binding by a net of hydrogen interactions between O6 and H520 (2.8 Å), D587 (3.0 Å), D207 (2.6 Å) and sodium ion (2.4 Å); O4 and D207 (2.6 Å); O3 and E517 (2.6 Å); O2 and M481 (3.3 Å) and Q441 (2.6 Å) (Figure 2).

**Figure 3:**
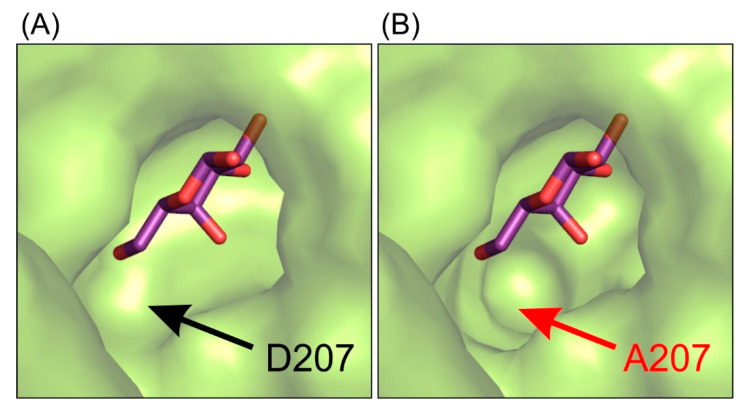
The representation of the shape of active site of *Arth*βDG_E441Q/gal complex structure (**A**) and superposition of galactose molecule on the structure of *Arth*βDG_D207A (**B**) showing that this mutation introduce changes in the shape of active site, making it less selective for galactosyl moiety.

This interaction pattern between the galactosyl moiety bound in the deep mode in the enzyme’s active site remains uninterrupted. Single-point mutations of residues E441 and E517 introduced in the enzyme’s active site were not sufficient to change spatial arrangement of galactosyl moiety bound in the deep binding mode but were enough to abolish the enzyme’s activity. Even though the mutant *Arth*βDG_D207A retained partial activity of the wild-type enzyme, no crystal structure of its complex with galactose bound within the binding site was obtained. The complex structure *Arth*βDG_D207A/gal showed a galactose bound only in G1 and G2 sites (galactose binding sites described in detail in part 2.5). Lack of even a residual electron density that could be attributed to galactose molecule presence in the active sites was consistent for more than 15 datasets obtained under different soaking conditions in triplicates. This may indicate that even though D207 is not a catalytic residue, its presence is essential for the enzyme function. D207 assures a correct position of the substrate by retaining the net of interactions and ensuring proper shape of the active site (Figure 3).

Combining the structural data with results of activity assays suggests that the mutation of D207 effects the enzyme’s activity by disturbing the hydroxyl group O4 stabilization of galactosyl moiety. It is caused by the cumulative effect of change in the shape of active site (Figure 1) and removing possible stabilizing contacts between carboxyl group of D207 and hydroxyl O4 of galactosyl moiety.

### 2.5. Galactose Binds on the Enzyme’s Surface

**Figure 4:**
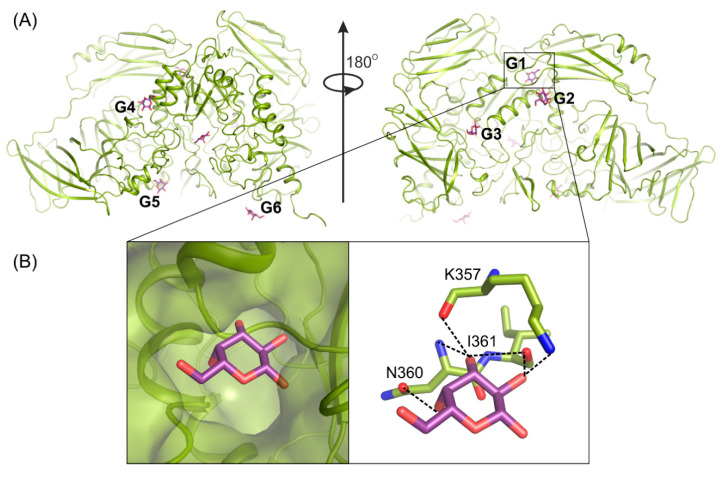
Galactose (violet) binding sites G1–G6 on the surface of *Arth*βDG (**A**); structure of *Arth*βDG_E441Q in complex with galactose (**B**). The galactose molecule (violet) interacting with main chain atoms at G1 site; galactose hydroxyl group O2 interacts with oxygen K357 (2.8 Å), oxygen I361 (2.9 Å); O3 with oxygen I361 (3.4 Å), nitrogen N360 (3.5 Å) and oxygen K357 (2.7 Å); O4 with N360 (2.8 Å).

Determining the crystal structure of *Arth*βDG_E441Q/gal complex at atomic resolution, 1.5 Å, resulted in discovering six galactose binding sites at the surface of the protein (Figure 4A). Five of these (G2–G6) have a potential to bind larger galactosides, e.g., lactose. The question, why such a large enzyme was developed and retained by extremophilic bacteria for catalysis of relatively simple reactions, remains open. However such a strategy, to elevate the accessibility of a substrate by its weak binding on the enzyme’s surface, may be one of explanation together with the maximization of the energy gain from the surface residue–solvent interactions [52].

Previously obtained biochemical data showed that d-galactose, one of the products of enzyme’s hydrolytic activity, has an inhibitory effect on protein’s activity. Its presence at 50 mM concentration results in the reduction of the enzyme’s activity by half [50]. The analysis of atomic resolution *Arth*βDG_E441Q/gal complex revealed that galactose binding site G1 is a possible allosteric site of enzyme. What is worth noting, is the addition of galactose was used for soaking of multiple *Arth*βDG complex structures to inhibit the activity of enzyme and, thus, enabled complex formation. Structural analysis of these complex structures showed that the galactose molecule was present at the G1 site for all of them, namely, previously published *Arth*βDG_E441Q/LACd [51] and, discussed here, *Arth*βDG_E517Q/lact and *Arth*βDG_ E441Q/lact. It should be stated that such a complex could not be successfully obtained without galactose addition.

The G1 site is located in the cleft formed at the junction of tree domains: Domain 2, 3, and 4 (Figure 4A). It is highly selective toward galactose molecules as residues N360, I361, and K357, constituting its bottom, shape it in such a way that binding of galactosyl moiety is highly preferred. The correct conformation of hydroxyl groups O2, O3, O4, and O6 is necessary for monosaccharide molecule to enter and bind within G1 site. Its limited size prevents basically any molecule larger than galactose from entering (Figure 4B).

### 2.6. ArthβDG Mutants in Complexes with Lactulose

**Figure 5:**
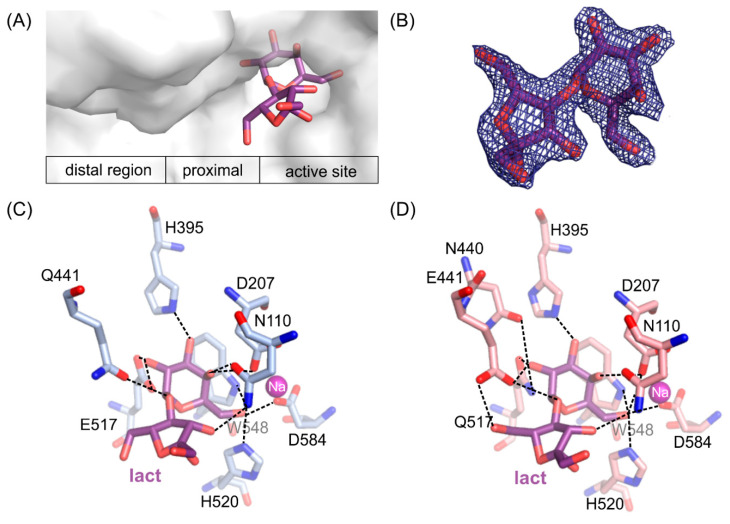
The position of lactulose binding by the loss of function mutants (**A**). *2F_o_-F_c_* electron-density map at the 2 σ level for the lactulose molecule in *Arth*βDG_E517Q/lact complex (**B**). Lactulose bound in the active site of *Arth*βDG_E441Q/lact (**C**) and *Arth*βDG_E517Q/lact (**D**) crystal structures with hydrogen bonds marked with dashed lines.

Crystal structures of loss of function *Arth*βDG_E441Q and *Arth*βDG_E517Q mutants in complexes with lactulose were determined both at 2.0 Å resolution. Lactulose molecule, a heterooligosaccharide composed of d-galactose and d-fructose linked through a β-(1→4)-glycosidic bond and is one of the products of transglycosylation reaction catalyzed by *Arth*βDG. It was bound in a deep mode in both structures (Figure 5A). The galactosyl moiety is stabilized by a net of interaction characteristic for binding of this group by *Arth*βDG, and described in detail in Section 2.4.

Fructose moiety of lactulose was stabilized relatively weakly in the position. In the case of a complex *Arth*βDG_E441Q/lact (Figure 5C), there was only one hydrogen bond between hydroxyl group O3′ of lactulose and N110 (3.2 Å). Whereas, in the case of *Arth*βDG_E517Q/lact (Figure 5D) there were two hydrogen bonds stabilizing fructose moiety: between O3′ of lactulose and N110 (3.3 Å) and O6’ and E441 (3.0 Å).

What is interesting, regardless of the relatively weak stabilization of the fructose moiety of lactulose, it creates more interactions within the active site of *Arth*βDG than glucosyl moiety of lactose, natural substrate [51]. The hydrogen bonds, forming between sugar moiety and the amino acids at the entrance to the active site, are crucial for sugar ring stabilization indispensable for it to become a galactosyl group acceptor. Such a delicate net of interactions suggests that residues E441, N110, and possibly N440 play important roles regarding transglycosylation activity of *Arth*βDG.

Insight into these two complex structures enables description of positioning and binding of fructose moiety, which may act as galactosyl group acceptor, in the catalytic center of *Arth*βDG mutants: *Arth*βDG_D207A and *Arth*βDG_E517Q. As both these mutations are distal to space occupied by fructose moiety, one may assume that similar interactions are in place in the case of wild-type enzyme. However, the substantially higher activity of wild-type enzyme makes it impossible to capture lactulose molecule bound in its active site.

### 2.7. Mapping the Binding Potential of the Distal Region of Active Site

**Figure 6:**
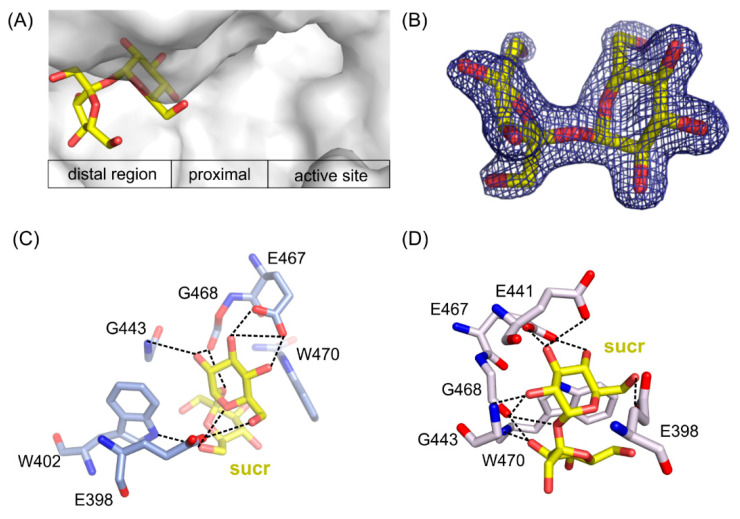
Location of sucrose molecule in the distal region of the active site in *Arth*βDG_E441Q/sucr complex (**A**). 2F_o_-F_c_ electron-density map at the 2 σ level for the saccharose molecule in *Arth*βDG_E441Q/sucr complex (**B**). Sucrose bound in the active site of *Arth*βDG_ E441Q/sucr (**C**) and *Arth*βDG_ D207A/sucr (**D**) crystal structures with hydrogen bonds marked with dashed lines.

Two structures of the mutants’ complexes with sucrose, a common sugar composed of d-galoctose and d-fructose linked through a β-(1→2)-glycosidic bond, were determined with resolution 1.7 Å for *Arth*βDG_E441Q/suc and 2.2 Å for *Arth*βDG_D207A/sucr. In both cases, sucrose was bound in the same place outside of the active site of enzyme, revealing a glucopyranose binding site in its distal region (Figure 6A).

In *Arth*βDG_D207A/sucr complex (Figure 6D), the sucrose molecule was stabilized predominantly by bonds between the glucosyl moiety interacting with residues E398, E467, E468, and G443. Fructosyl moiety was stabilized by only two hydrogen bonds created between hydroxyl O6’ and side chain oxygen of E398 (2.5 Å) and the same hydroxyl group O6’ with nitrogen of W402 indole ring (3.1 Å).

Sucrose molecule in complex structure of *Arth*βDG_E441Q/sucr moved a little toward the active site of enzyme (Figure 6C), and its hydroxyl 03 interacted via hydrogen bond with the mutated catalytic residue Q441 (3.1 Å). Nonetheless, it is stabilized by a similar net of interactions involving residues E398, E467, G443, and additionally E398. Hydroxyl group O3′ of fructosyl moiety was stabilized by a single hydrogen bond with indole ring nitrogen of W470.

**Figure 7:**
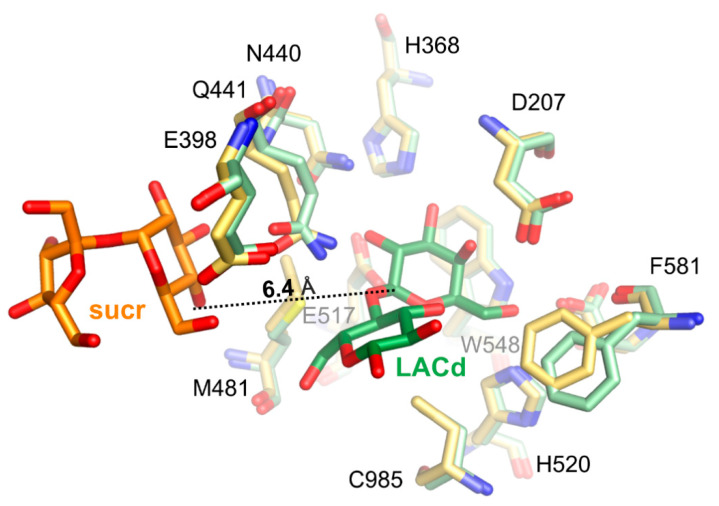
Superposition of *Arth*βDG_E441Q/sucr (yellow/orange) with complex structure of the same mutant with lactose bound in deep mode (pale green/green), *Arth*βDG_E441Q/LACd (PDB ID: 6SEA), showing the positioning of sucrose molecule toward galactosyl moiety.

Superposition of *Arth*βDG_E441Q/sucr with complex structure of the same mutant with lactose bound in deep mode, *Arth*βDG_E441Q/LACd (PDB ID: 6SEA [51]) shows that the distance between anomeric carbon atom of galactosyl moiety bound in deep mode and hydroxyl group O4 of sucrose was 6.4 Å (Figure 7). This insight into sucrose binding in the distal region of active site shows how the glycosyl moiety is positioned in the vicinity of the active site, so it can become galactosyl group acceptor.

## 3. Summary

Results of activity assays and analysis of a number of complex structures of new mutants of *Arth*βDG, *Arth*βDG_D207A and *Arth*βDG_E517Q, resulted in a better understanding of the importance of residues D207, E441, and E517 in the native enzyme. The inactive mutant E517Q was designed specially to enable binding of transglycosylation product—lactulose, in the active site of enzyme and thus elucidating the mode of its binding. Especially surprising was the gravity of mutation of D207 to alanine, which is part of Domain 1. It is situated on the top of a part of Domain 1, which inserts itself in a space between the top of Domain 3 TIM-barrel and highly flexible C-terminal region of Domain 3, and constitutes bottom of the active site, which means that its mutation to alanine has a significant impact on active site volume. As such, not only a stabilizing interaction between D207 and O4 hydroxyl of galactosyl moiety is lost but also the transfer of substrate from shallow to deep binding site of the enzyme is disrupted.

**Figure 8:**
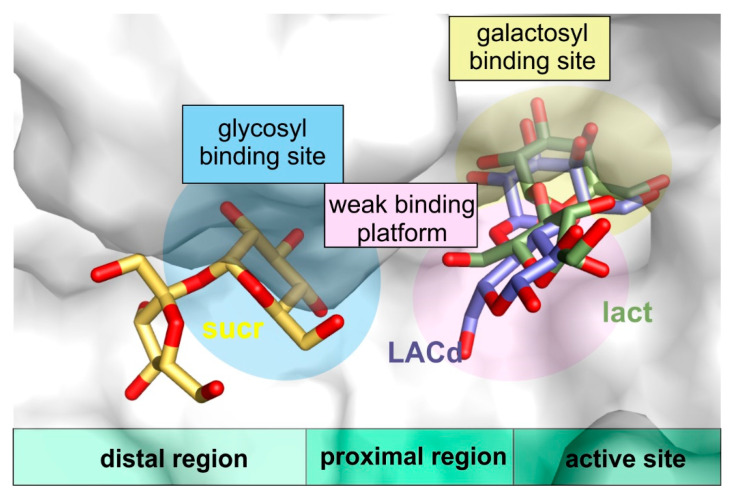
The visualization of proximal region (weak binding platform) and distal region of active site (with glycosyl binding site) in superposed complex structures of *Arth*βDG_E441Q/sucr, *Arth*βDG_E441Q/LACd, and *Arth*βDG_E517Q/lact.

Furthermore, the structures of *Arth*βDG_D207A and *Arth*βDG_E517Q in complex with lactulose, which can be produced by *Arth*βDG in course of transglycosylation reaction, were, to best of our knowledge, the first crystal structures of galactosidase with this heterooligosaccharide, thus, providing a sought for insight in how a galactosyl group acceptor (larger than water) may approach and be accommodated (Figure 8). Such a detailed structural knowledge on the binding of lactulose pinpoints the residues that play role in the production of lactulose by *Arth*βDG, especially: N110. It also shows the region, where some additional hydrogen bonds, stabilizing fructosyl moiety, can be introduced to shift the reaction toward formation of lactulose instead of simple hydrolysis of glucose.

More valuable structural information that concerns transglycosylation activity of the enzyme was obtained with a solution of the complex structures with sucrose, *Arth*βDG_D207A/sucr and *Arth*βDG_E441Q/sucr. They revealed a glucopyranose binding site in the distal region of active site, showing how a galactosyl group acceptor that comprises of glucosyl moiety can approach the active site. Once again, these structures elucidate the region, mutation in which can lead to larger galactosyl group acceptor being available instead of water residue.

The results of this work can serve as a basis for knowledge-based enzyme engineering of this cold-adapted enzyme. Especially, to shift its activity toward transglycosylation reaction and improving product homogeneity. It was shown that the retention of the shape and volume of the galactosyl binding site is necessary for the enzyme’s activity. Hence, mutations, if any introduced in this region should be designed in such a way not to change the size of deep binding site, as its enlargement not only does not enhance the enzyme’s transglycosylation activity but is has a negative effect on it. It is the authors’ believe that mutations should rather be introduced in the proximal in distal regions of the active site. Especially, promoting binding of galactosyl group acceptor on the weak binding platform may prove successful in the future attempts.

Furthermore, since the architecture of the active site of galactosidases from GH2 family is highly conserved, the knowledge about the mode of binding of potential galactosyl group acceptors (larger than water) within *Arth*βDG and its mutant structures, can be to a certain degree transferred onto other enzymes belonging to this group, among others commonly used in the food industry β-d-galactosidase from *Kluyveromyces lactis*.

## 4. Materials and Methods

### 4.1. Site-Directed Mutagenesis of Gene Encoding ArthβDG

The gene encoding the *Arth*βDG enzyme within the pBAD-Bgal32cB plasmid [50] has been mutated using the Q5 Site-Directed Mutagenesis Kit (New England Biolabs, Ipswich, MA, USA) following the manufacturer’s protocol. For this purpose, two pairs of mutagenic primers were designed and synthesized (Genomed, Warsaw, Poland). Primers For207AspAla 5′ GTGGAGGACCAGG**C**CATGTGGTGGCTT 3′ and Rev207AspAla 5′ GTAGCTGGCGGCCGAGAACTGGGCGA 3′ were used to introduce a point mutation A/C at 620 nucleotide position in the gene resulting in the D207A substitution in the *Arth*βDG amino acid sequence. Primers F32cBmut517 5′ TGGGTAACGGCCCCGGTGGAATGAGCGAAT 3′ and R32cBmut517 5′ TGGCATGCACATATT**G**GCAGAGGACAAAGGGCA 3′ were used to introduce a point mutation G/C at 1549 nucleotide position in the gene resulting in the E517Q substitution in the *Arth*βDG amino acid sequence.

Thermocycling conditions for PCRs were as follows: initial denaturation of pBAD-Bgal32cB plasmid at 98 °C for 30 s; then 25 cycles of PCR products amplification consisting of 10 s of DNA denaturation at 98 °C, 15 s of mutagenic primers annealing at 66 °C for For207AspAla and Rev207AspAla, or 69 °C for F32cBmut517 and R32cBmut517, and 3 min 20 s of PCR product extension at 72 °C; and the final extension at 72 °C for 7 min.

The obtained PCR products were treated with KLD Enzyme Mix (Kinase, Ligase, and *Dpn*I) at 22 °C for 5 min, and then used to transform NEB 5-alpha chemically competent *E.coli* cells (the *lac*Z deletion mutant, D (*lac*Z) M15). Transformants were spread onto selection Luria-Bertani agar plates (10 g/L of peptone K, 5 g/L of yeast extract, 10 g/L of NaCl, and 15 g/L of agar) supplemented with ampicillin (100 μg/mL), X-gal (40 μg/mL) and l-arabinose (200 μg/mL), and incubated overnight at 37 °C and then for a few hours at room temperature.

Six colonies of *E. coli* + pBAD-Bgal32cB_D207A (light blue colonies with weak β-d-galactosidase activity) and *E. coli* + pBAD-Bgal32cB_E517Q (white colonies without enzymatic activity) were selected for further studies. Recombinant pBAD-Bgal32cB_D207A and pBAD-Bgal32cB_E517Q plasmids were isolated using the Plasmid Mini Kit (A&A Biotechnology, Gdynia, Poland) and sequenced (Genomed, Warsaw, Poland).

### 4.2. Production of ArthβDG, ArthβDG_D207A and ArthβDG_E517Q Proteins

Expression of recombinant *Arth*βDG, *Arth*βDG_D207A, and *Arth*βDG_E517Q mutants were performed in the *E. coli* LMG 194 cells (Invitrogen, Carlsbad, CA, USA) transformed with pBAD-Bgal32cB, pBAD-Bgal32cB_D207A and pBAD-Bgal32cB_E517Q plasmids, respectively, as previously described for wild-type protein [50] and *Arth*βDG_E441Q mutant [51].

Recombinant proteins were purified by a three-step chromatography comprising of ion-exchange and size-exclusion stages according to protocol developed for wild-type protein, *Arth*βDG [7]. The chromatography buffer was change to storage buffer (0.05 M HEPES pH 7.0) and the samples were concentrated to 20 mg/mL using 50 kDa cutoff membrane Vivaspin filters (Sartorius, Göttingen, Germany) prior to crystallization.

### 4.3. Determination of ArthβDG, ArthβDG_D207A and ArthβDG_E517Q Activity

The activity of *Arth*βDG as well as *Arth*βDG_D207A and *Arth*βDG_E517Q mutants was determined using ONPG as a substrate. 0.8 mL of ONPG solution (1 mg/mL in 20 mM potassium phosphate buffer pH 8.0) was preincubated at 28 °C for 5 min, then 0.2 mL of protein sample was added, and the reaction mixture was incubated for 1 or 10 min at 28 °C for the substrate hydrolysis. The reaction was terminated by the addition of 0.3 mL of 1 M Na_2_CO_3_ and the absorbance was measured at 410 nm. One unit of β-d-galactosidase activity was defined as the quantity of enzyme releasing of 1 μmol 2-nitrophenol per min under reaction conditions.

### 4.4. Thermofluor Shift Assay

Samples of *Arth*βDG_D207A and *Arth*βDG_E517Q were prepared at concentration of 0.3 mg/mL each and premixed with SYPRO. The CFX96 Touch^TM^ (BioRad, Hercules, CA, USA) thermal cycles was used for TSA experiment. The applied temperature increment was 1 °C per 30 s, and the tested temperature ranged from 4 °C to 95 °C. The assay was performed for 24 buffers covering pH range from 4.0 to 10.0 and 24 corresponding buffers with addition of 250 mM NaCl [54].

### 4.5. Crystallization of ArthβDG Mutants and Obtaining Their Complexes’

Crystallization of *Arth*βDG_D207A, *Arth*βDG_E441Q and *Arth*βDG_E517Q was performed by diffusion hanging-drop method using Tacsimate^TM^ Hampton Research (Aliso Viejo, CA, USA) at concentration range from 27% to 45% and pH range from 4.0 to 9.0 [7]. This crystallization matrix was previously used for growing crystals of wild-type enzyme. Furthermore, the cross-seeding using seeds made of *Arth*βDG crystals was applied to speed up the process of obtaining diffraction quality crystals. The seed stock of crushed microcrystals diluted 10,000 times in 35% Tacsimate^TM^ pH 7.0 was premixed in 1:40 (*v/v*) ratio with 20 mg/mL purified protein according to previously established protocol [51]. The monocrystals suitable for diffraction experiment were obtained in a pH range of 6.4–8.8 and Tacsimate^TM^ concentration varying from 24% to 40%.

The complex *Arth*βDG_E517Q/gal was obtained after 24 h soaking of protein crystals with addition of globotriose and *Arth*βDG_D207A/gal by soaking with addition of mixture of galactose and fructose. A 24h-soaking was successful in the case of complexes *Arth*βDG_D207A/lact and *Arth*βDG_E517Q/lact, where the crystals were soaked with addition of a mixture of lactulose and galactose. The complexes of *Arth*βDG_E441Q and *Arth*βDG_D207A with saccharose were obtained by same time soaking with addition of saccharose alone.

All the crystals were cryo-protected with 60% Tacsimate^TM^ of pH corresponding to crystallization conditions prior to being flash-frozen before diffraction experiment.

### 4.6. Data Collection, Structure Solving, and Refinement

High-resolution diffraction data were collected using state-of-the-art BESSY II beamlines 14.1 and 14.2, Berlin, Germany [55]. The diffraction images were collected with fine slicing 0.1°. The diffraction data were processed using XDSapp [56], to avoid alternative indexing, possible in space group P3_1_21, the log file for processing *Arth*βDG data was used as an input. Pair refinement was performed to determine optimal cutoff resolution for each data set. Crystal structures were solved by isomorphous replacement using rigid body refinement procedure, where the structure of *Arth*βDG (PDB ID: 6ETZ) was used as a model. Structure solving and further refinement was performed using the PHENIX.REFINE program [57,58] data reduction and refinement statistics are collected in Table 2 for *Arth*βDG_E517Q, Table 3 for *Arth*βDG_E441Q, and Table 4 for *Arth*βDG_D207A.

### 4.7. Databases

The here reported crystal structures and their associated structure factor amplitudes were deposited with the Protein Data Bank under the accession codes: 6ZJP, 6ZJQ, 6ZJR, 6ZJS, 6ZJT, 6ZJU, 6ZJV, 6ZJW AND 6ZJX respectively.

## Figures and Tables

**Table 1:** Activity of *Arth*βDG, *Arth*βDG_D207A, and *Arth*βDG_E517Q in hydrolysis reaction. The activity was measured with ONPG as a substrate at 28 °C and pH 8.0.

Protein	Specific Activity (U/mg)
*Arth*βDG	212.01 ± 3.90
*Arth*βDG_D207A	0.70 ± 0.01
*Arth*βDG_E517Q	0

**Table 2:** Data reduction and refinement statistics of *Arth*βDG_E517Q mutant’s crystal structures.

Crystal Structure	*Arth*βDG_E517Q PDB ID: 6ZJP	*Arth*βDG_E517Q/gal PDB ID: 6ZJQ	*Arth*βDG_E517Q/lact PDB ID: 6ZJR
Diffraction source	BL 14.2 BESSY, Berlin, Germany	BL 14.2 BESSY, Berlin, Germany	BL 14.1 BESSY, Berlin, Germany
Wavelength (Å)	0.918400	0.918400	0.918400
Temperature (K)	100 K	100 K	100 K
Detector	PILATUS 3S 2M	PILATUS 3S 2M	PILATUS 3S 2M
Rotation range per image (°)	0.1	0.1	0.1
Total rotation range (°)	100	180	180
Exposure time per image (s)	0.2	0.2	0.2
Space group	P 31 2 1	P 31 2 1	P 31 2 1
*a*, *b*, *c* (Å)	138.0 138.0 127.2	138.6 138.6 127.7	137.4 137.4 127.3
α, β, γ (°)	90 90 120	90 90 120	90 90 120
Mosaicity (°)	0.13	0.09	0.09
Resolution range (Å)	43.6–1.8 (1.9–1.8)	42.8–1.7 (1.8–1.7)	46.7–2.0 (2.1–2.0)
Number of unique reflections	118,415 (11695)	155,098 (15193)	93,953 (9191)
Completeness (%)	99.08 (98.40)	99.85 (98.78)	99.76 (99.06)
Redundancy	5.1	10.2	10.1
I/σ(I)	12.39 (1.52)	13.5 (0.6)	12.54 (0.78)
*R*_meas_ (%)	7.5 (81.7)	13.6 (380.9)	15.6 (257.7)
Overall *B* factor: Wilson plot/refinement (Å^2^)	31.06	28.08	38.62
No. of reflections: working/test set	118,262/2096	155,074/2099	93,830/2097
*R*/*R*_free_	0.1691/0.1946	0.1729/0.2008	0.1881/0.2131
No. of non-H atoms: Protein/Ligand/Water	7613/7/1050	7624/151/621	7702/79/528
R.m.s. deviations: Bonds (Å)/Angles (°)	0.005/0.74	0.010/1.00	0.006/0.79
Ramachandran plot: Most favored/allowed (%)	97.06/2.94	97.06/2.94	96.96/3.04

Values in parenthesis are given for highest resolution shell.

**Table 3:** Data reduction and refinement statistics of *Arth*βDG_E441Q mutant’s crystal structures.

Crystal Structure	*Arth*βDG_E441Q/gal PDB ID: 6ZJS	*Arth*βDG_E441Q/lact PDB ID: 6ZJT	*Arth*βDG_E441Q/sucr PDB ID: 6ZJU
Diffraction source	BL 14.2 BESSY, Berlin, Germany	BL 14.2 BESSY, Berlin, Germany	BL 14.2 BESSY, Berlin, Germany
Wavelength (Å)	0.918400	0.918400	0.918400
Temperature (K)	100 K	100 K	100 K
Detector	PILATUS 3S 2M	PILATUS 3S 2M	PILATUS 3S 2M
Rotation range per image (°)	0.1	0.1	0.1
Total rotation range (°)	180	100	180
Exposure time per image (s)	0.2	0.2	0.3
Space group	P 31 2 1	P 31 2 1	P 31 2 1
*a*, *b*, *c* (Å)	138.4 138.4 127.8	139.0 139.0 127.7	138.2 138.2 127.6
α, β, γ (°)	90 90 120	90 90 120	90 90 120
Mosaicity (°)	0.03	0.11	0.18
Resolution range (Å)	45.3–1.7 (1.8–1.7)	43.8–2.0 (2.1–2.0)	43.7–1.7 (1.8–1.7)
Number of unique reflections	224,735 (22177)	100,340 (9948)	141,152 (13911)
Completeness (%)	99.91 (99.29)	99.49 (99.26)	99.87 (99.42)
Redundancy	10,1	5.6	10.1
I/σ(I)	10,1 (0,6)	12.14 (0.80)	14.42 (0.56)
*R*_meas_ (%)	13,0 (350,5)	7.9 (175.3)	13.6 (425.6)
Overall *B* factor: Wilson plot/refinement (Å^2^)	25.29	45.01	30.65
Number of reflections: working/test set	224,710/2358	100,137/2096	141,107/2099
*R*/*R*_free_	0.1550/0.1706	0.2100/0.2362	0.1786/0.1941
Number of non-H atoms: Protein/Ligand/Water	7662/205/721	7615/80/337	7627/99/751
R.m.s. deviations: Bonds (Å)/Angles (°)	0.008/0.99	0.009/0.92	0.011/1.17
Ramachandran plot: Most favored/allowed (%)	97.87/2.12	96.96/2.94	97.16/2.84

Values in parenthesis are given for highest resolution shell.

**Table 4:** Data reduction and refinement statistics of *Arth*βDG_D207A mutant’s crystal structures.

Crystal Structure	*Arth*βDG_D207A PDB ID: 6ZJV	*Arth*βDG_D207A/gal PDB ID: 6ZJW	*Arth*βDG_D207A/sucr PDB ID: 6ZJX
Diffraction source	BL 14.2 BESSY, Berlin, Germany	BL 14.2 BESSY, Berlin, Germany	BL 14.2 BESSY, Berlin, Germany
Wavelength (Å)	0.918400	0.918400	0.918400
Temperature (K)	100 K	100 K	100 K
Detector	PILATUS 3S 2M	PILATUS 3S 2M	PILATUS 3S 2M
Rotation range per image (°)	0.1	0.1	0.1
Total rotation range (°)	360	100	180
Exposure time per image (s)	0.2	0.2	0.2
Space group	P 31 2 1	P 31 2 1	P 31 2 1
*a*, *b*, *c* (Å)	139.5 139.5 127.9	139.3 139.3 127.8	137.6 137.6 126.8
α, β, γ (°)	90 90 120	90 90 120	90 90 120
Mosaicity (°)	0.17	0.30	0.14
Resolution range (Å)	47.1–2.2 (2.3–2.2)	40.2–2.1 (2.2–2.1)	46.6–2.2 (2.3–2.2)
Number of unique reflections	68,432 (6677)	81,059 (7962)	69,828 (6808)
Completeness (%)	99.25 (97.39)	99.35 (98.55)	99.72 (98.12)
Redundancy	20.0	5.4	10.0
I/σ(I)	21.38 (1.52)	5.18 (0.54)	12.58 (1.28)
*R*_meas_ (%)	11.4 (184.8)	19.9 (200.6)	14.8 (168.7)
Overall *B* factor: Wilson plot/refinement (Å^2^)	50.40	46.09	42.43
Number of reflections: working/test set	68,233/1094	80,908/2083	69,783/1116
*R*/*R*_free_	0.2361/0.2623	0.2258/0.2608	0.1971/0.2238
Number of non-H atoms: Protein/Ligand/Water	7690/7/142	7620/24/303	7622/58/530
R.m.s. deviations: Bonds (Å)/Angles (°)	0.005/0.84	0.003/0.66	0.004/0.66
Ramachandran plot: Most favored/allowed (%)	97.26/2.74	96.56/3.44	96.96/3.04

Values in parenthesis are given for highest resolution shell.

## Supplementary Materials

The following are available online at https://www.mdpi.com/1422-0067/21/15/5354/s1, Figure S1: Selected TSA data and analysis for *Arth*βDG_D207A mutant, Figure S2: Selected TSA data and analysis for *Arth*βDG_E517Q mutant.

## Abbreviations

ArthβDG	β-d-galactosidase from *Arthrobacter* sp. 32cB
*Arth*βDG_D207A	β-d-galactosidase from *Arthrobacter* sp. 32cB mutant D207A
*Arth*βDG_E441Q	β-d-galactosidase from *Arthrobacter* sp. 32cB mutant E441Q
*Arth*βDG_E517Q	β-d-galactosidase from *Arthrobacter* sp. 32cB mutant E517Q
Gal	galactose
GOS	galactooligosaccharides
GH2	glycosyl hydrolase 2 family
HOS	heterooligosaccharides
Lacd	lactose bound in deep mode
Lact	lactulose
ONPG	*o*-nitrophenyl-β-d-galactopyranoside
Sucr	sucrose
TSA	thermofluor shift assay
