# Supplementary material for: Mapping the Transglycosylation Relevant Sites of Cold-Adapted β-d-Galactosidase from Arthrobacter sp. 32cB
Source: Int J Mol Sci. 2020 Jul 28;21(15):5354. doi: 10.3390/ijms21155354 (PMC7432029; doi:10.3390/ijms21155354)
Supplement: Supplementary file 1 [file ijms-21-05354-s001.pdf]

Supplementary materials

(A)

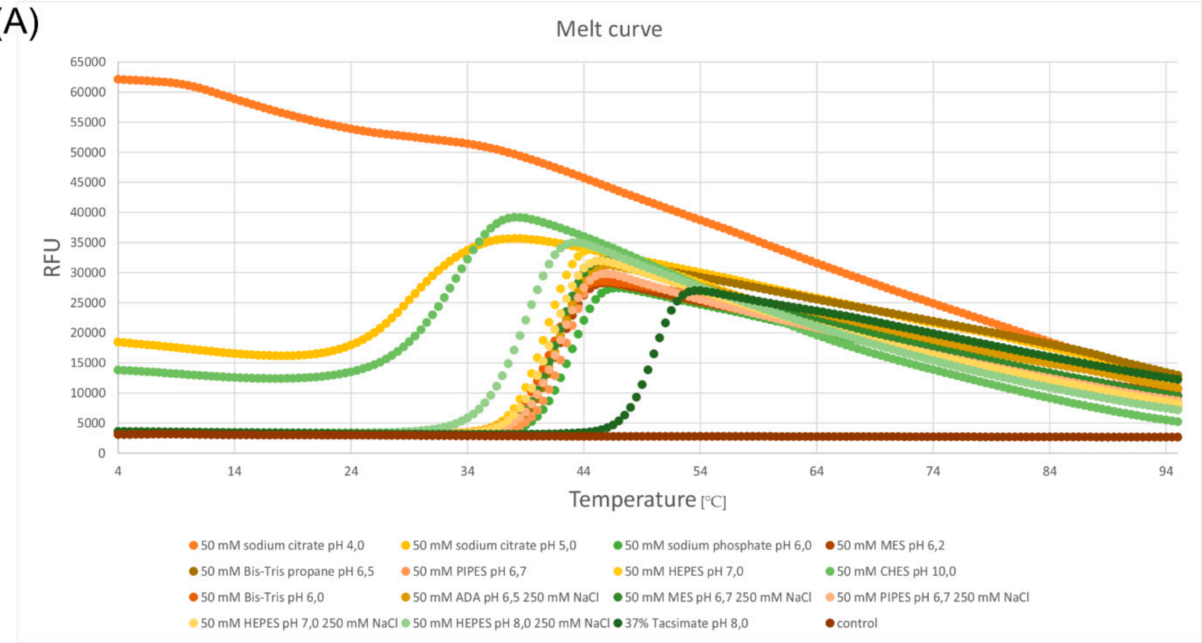

(B)

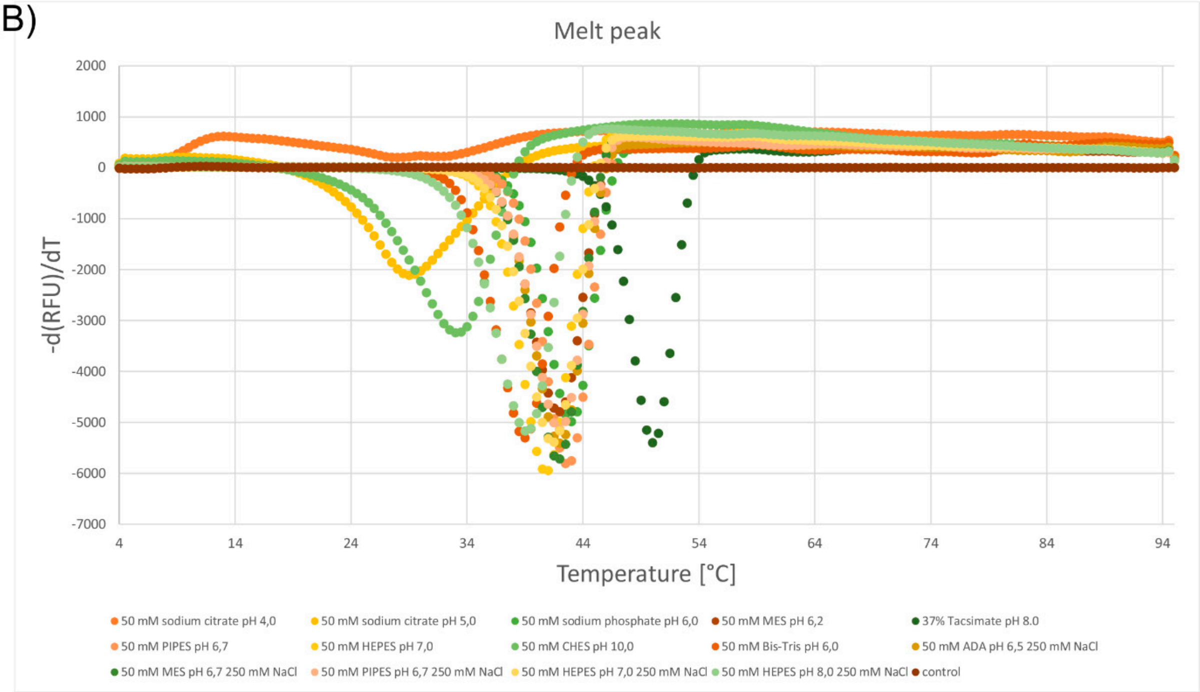

Figure S1. Selected TSA data and analysis for ArthβDG_D207A mutant. (A) Thermofluor-base protein-unfolding curves of ArthβDG_D207A. (B) The melting peak T_m identified by plotting the first derivative of the fluorescence emission as a function of temperature (-d(RFU)/dT).

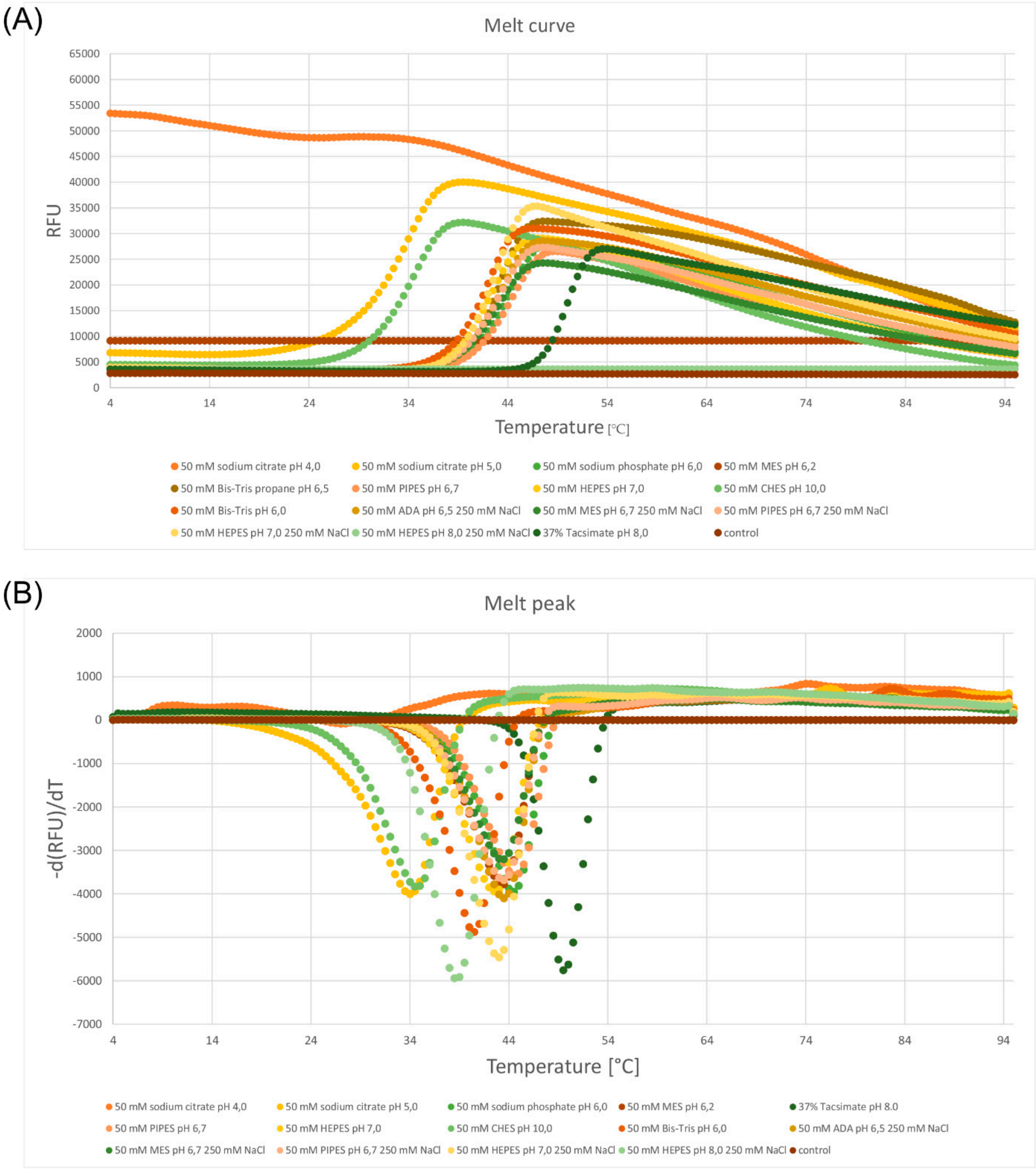

Figure S2. Selected TSA data and analysis for ArthβDG_E517Q mutant. (A) Thermofluor-base protein-unfolding curves of ArthβDG_ E517Q. (B) The melting peak T_m identified by plotting the first derivative of the fluorescence emission as a function of temperature (-d(RFU)/dT).
